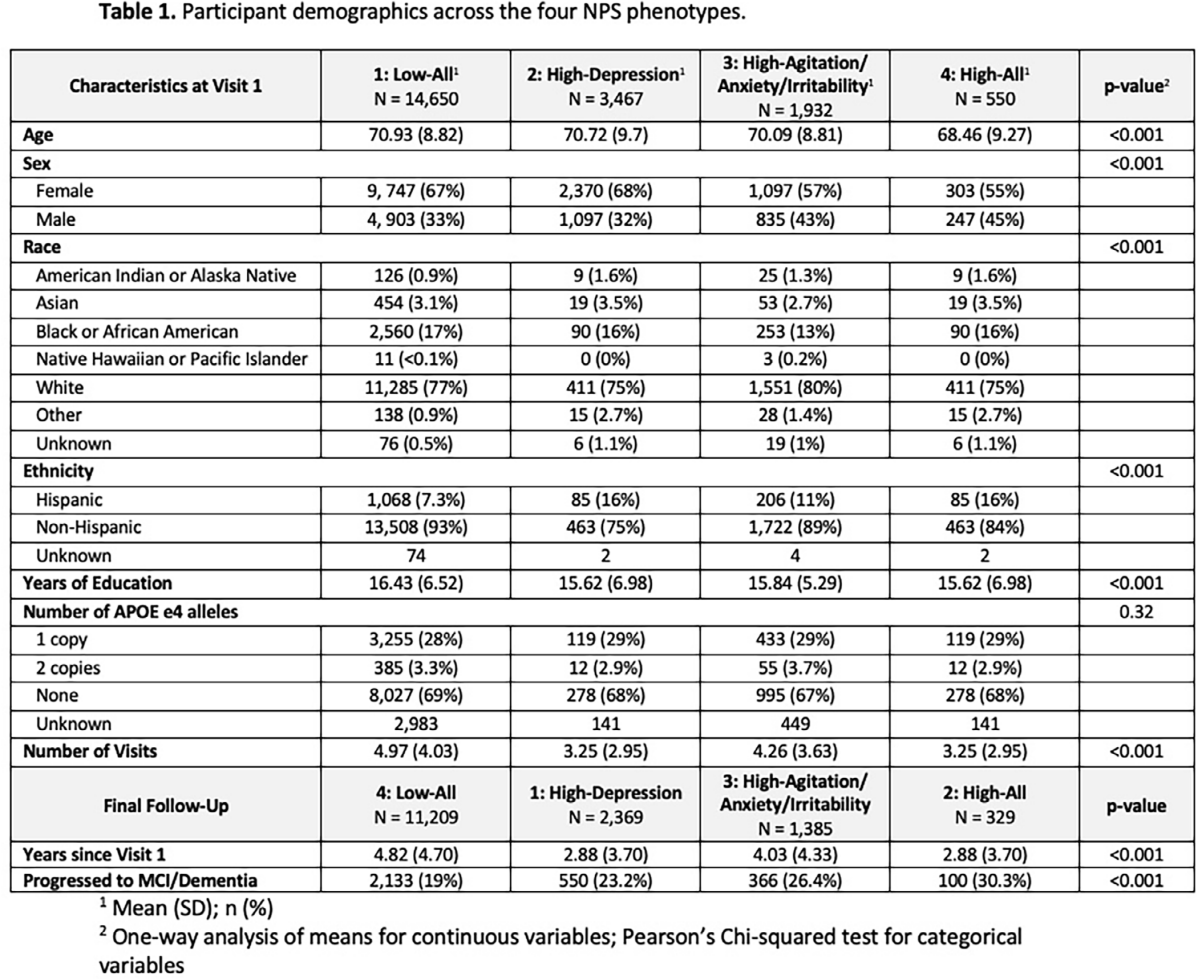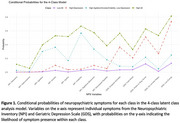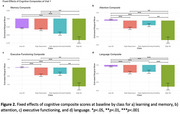# Neuropsychiatric Phenotypes in Cognitively Unimpaired Older Adults: Associations with Clinical Progression and Domain‐Specific Cognitive Trajectories

**DOI:** 10.1002/alz70857_097877

**Published:** 2025-12-24

**Authors:** Fareshte Erani, Caitlin M. Terao, Shanna Cooper, Alexandra J. Weigand, Katherine J. Bangen, Emily C. Edmonds, Kelsey R. Thomas

**Affiliations:** ^1^ University of California, San Diego, La Jolla, CA, USA; ^2^ San Diego State University/University of California San Diego Joint Doctoral Program in Clinical Psychology, San Diego, CA, USA; ^3^ Oregon Health and Science Unviersity, Portland, OR, USA; ^4^ University of California San Francisco, San Francisco, CA, USA; ^5^ VA San Diego Healthcare System, San Diego, CA, USA; ^6^ Banner Alzheimer's Institute, Tucson, AZ, USA; ^7^ University of Arizona, Tucson, AZ, USA

## Abstract

**Background:**

Data‐driven analyses of neuropsychological measures have identified subtle cognitive decline phenotypes that demonstrate unique associations with Alzheimer's disease risk and progression. While early detection efforts traditionally focus on cognitive and biological markers, neuropsychiatric symptoms (NPS) have emerged as critical predictors of cognitive decline and dementia conversion. The current study examined whether distinct NPS phenotypes were associated with progression to mild cognitive impairment (MCI)/dementia and cognitive decline trajectories.

**Method:**

A latent class analysis was conducted on 20,599 cognitively unimpaired (CU) older adults in the National Alzheimer's Coordinating Center Uniform Data Set identified NPS phenotypes using item‐level variables from the Neuropsychiatric Inventory and Geriatric Depression Scale. Logistic regressions with Tukey‐adjusted contrasts assessed odds of progression to MCI/dementia after adjusting for age, sex, education, and years since baseline. Linear mixed‐effects models, adjusting for demographics, examined 6‐year changes in cognitive composite scores (memory, attention, executive function, and language) by NPS phenotype.

**Result:**

The best‐fiting model had four NPS phenotypes: [1] Low‐All; [2] High‐Depression; [3] High‐Agitation/Anxiety/Irritability, and [4] High‐All (Table 1, Figure 1). The *High‐All* group had the greatest progression risk to MCI/dementia, with 3x higher odds than *Low‐All* (*p* < .001), 2.1x higher odds than *High‐Depression* (*p* < .001), and 1.6x higher odds than *High‐Agitation/Anxiety/Irritability* (*p* = .009). The *High‐Agitation/Anxiety/ Irritability* group had 2x higher odds than *Low‐All* (*p* < .001) and 1.4x higher odds than *High‐Depression*, while the *High‐Depression* group had 1.4x higher odds than *Low‐All*. At baseline, the *Low‐All* group outperformed others across cognitive domains (all *p*s < .001) except for memory, where *High‐Depression* did not differ (Figure 2). The *High‐Depression* group showed faster decline in attention than *Low‐All*, while the *High‐All* group declined faster in executive functioning (*p*s < .01). The *High‐Agitation/Anxiety/Irritability* group showed slower memory improvement (*p* = .003) and faster decline across all other cognitive domains (*p*s < .01) compared to the *Low‐All group*.

**Conclusion:**

Distinct NPS phenotypes were observed among CU older adults and were associated with differing risks of MCI/dementia progression and cognitive trajectories. Phenotypes with high NPS, particularly agitation, anxiety, and irritability, showed higher odds of progression than depressive symptoms alone. These findings underscore the value and potential utility of early NPS patterns for predicting cognitive decline and early risk detection.